# In Vivo Efficacy of an Adhesive Bioresorbable Patch to Treat Aortic Dissections

**DOI:** 10.1016/j.jacbts.2023.08.002

**Published:** 2023-10-11

**Authors:** Noemí Balà, Alejandro Aranda, Pau Teixidó, Carlota Molhoek, Inés Moreno-Jiménez, Germán Febas, Júlia López-Guimet, Adam Groothuis, Elazer Reuven Edelman, Mercedes Balcells, Salvador Borrós, Jordi Martorell, Vicente Riambau

**Affiliations:** a IQS School of Engineering, Universitat Ramon Llull, Barcelona, Spain; b Aortyx SL, Teia, Spain; c Institute for Medical Engineering and Sciences, Massachusetts Institute of Technology, Cambridge, Massachusetts, USA; d Vascular Surgery Department, Hospital Clínic de Barcelona, Barcelona, Spain

**Keywords:** aorta, biomaterials, dissection

## Abstract

Endovascular repair of aortic dissection still presents significant limitations. Preserving the mechanical and biological properties set by the aortic microstructure is critical to the success of implantable grafts. In this paper, we present the performance of an adhesive bioresorbable patch designed to cover the entry tear of aortic dissections. We demonstrate the power of using a biomimetic scaffold in a vascular environment.

## Highlights

• Two-faced polycaprolactone patches were electrospun. The luminal face consisted of aligned fibers designed to align with blood flow, to promote endothelial cell migration and to stop underlying smooth muscle cell colonization. The abluminal face of the patch was comprised of random fibers with high porosity, aimed at promoting smooth muscle cell colonization and macrophage clustering. This structure guarantees full integration within the native vascular tissue, promoting differential colonization by endothelial and smooth muscle cells.
• The aorta has viscoelastic properties, and these are not met by any available grafts or stents nowadays. Mimicking the aorta’s mechanical properties is critical to avoid mechanical mismatches and consequent device failure. Our mechanical testing indicates that the patch design is able to mimic those properties, and echocardiographic measurements validate that indeed we are in the range of natural tissue properties, because the patch is fully compliant with the natural motion of the aorta.
• Patch and adhesive biocompatibility was assessed in vivo and ex vivo. The patch-adhesive system failed to cause any thrombogenicity in standardized tests. Cytotoxicity was very low in vitro, and animal studies showed minimal to no inflammation and thrombogenicity up to 90 days in large animals. Together, these results show that neither the patch nor the adhesive compromise cell viability.

## Visual Abstract

**Figure:**
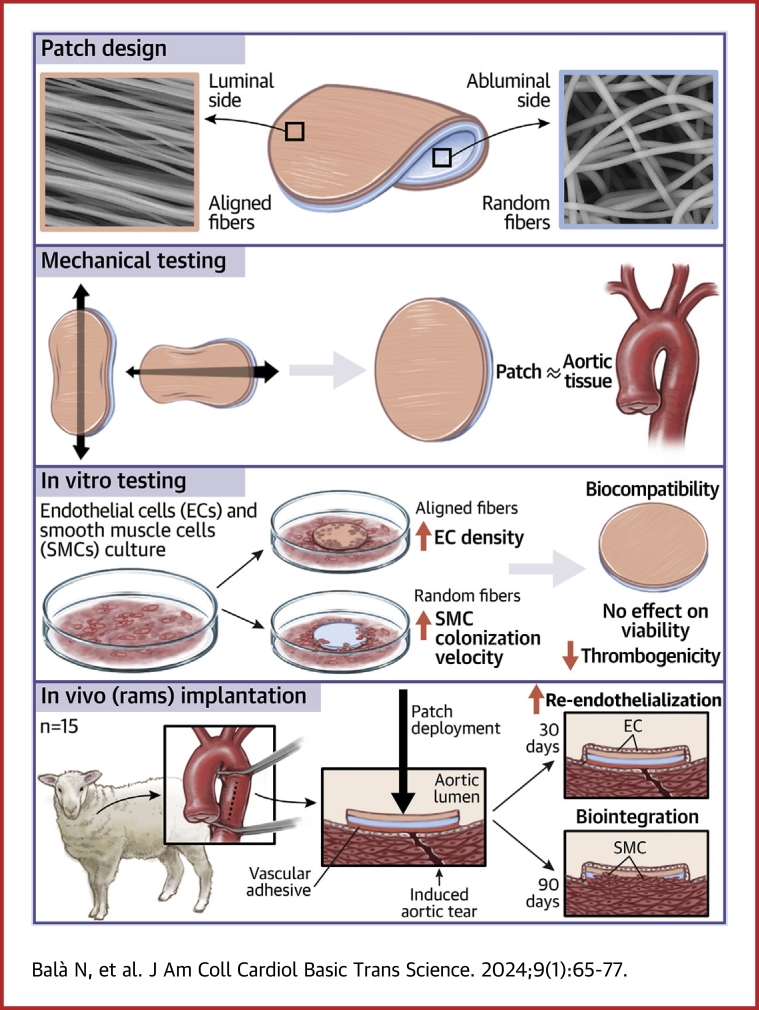

Aortic dissection, the tearing through the layers of the aortic wall, is the most frequent and catastrophic manifestation of the acute aortic syndrome.^1^ Its worldwide annual incidence is ∼10 in 100,000 patients, with a 67% male preponderance^2^ and a global mortality of up to 50%, depending on the dissection type and patient conditions.^3^^,^^4^ In the past, surgical replacement of the dissected section was the only effective treatment, carrying, however, high risks of mortality and morbidity.^5^^,^^6^ Numerous studies have reported the safety and efficacy of thoracic endovascular aortic repair, especially for type B aortic dissection.^7^ Endovascular interventions using stented grafts reduce mortality and length of hospitalization, but a large number of patients are ineligible for this type of intervention. Overall, stents have reported good results but still present significant limitations.^8^^,^^9^ On the negative side, these grafts do not fully conform to the patient’s aorta; their apposition is always complicated. If the stent is underexpanded, thrombi can form and/or the graft may be displaced over time. If the stent is overexpanded, it creates microinjuries along the lumen that further damage the already jeopardized vessel creating leaks and even rupture. Surgical and endovascular repair grafts use materials such as Dacron or Teflon whose increased stiffness causes mechanical mismatch between the natural tissue and the device,^10^ resulting in increased cardiac load and transmission of the pressure wave downstream. These grafts, inert and nondegradable, do not actively promote false lumen clotting and resorption, nor vascular remodeling and regeneration, preventing the vascular wall from fully recovering its function. This combination of events ultimately leads to secondary aneurysms and dissections,^11^ and thus, in the long-term, reintervention is very common.^12^, ^13^, ^14^ In this context, preserving the mechanical and biological properties set by the aortic microstructure is critical when designing medical devices aimed at treating aortic diseases.

The aortic microstructure consists primarily of 3 layers or tunics, the *intima*, *media*, and *adventitia*, populated mainly by endothelial cells, smooth muscle cells, and fibroblasts, respectively. An intact, confluent endothelium prevents the formation of blood clots, while smooth muscle cells and fibroblasts synthesize collagen and elastin that provide structure and strength and mediate macroscopic contractility and elasticity of the artery. In the aorta, elasticity is nonlinear because of its anisotropy.^15^ Even in the same segment, properties vary along the circumferential and longitudinal directions.^16^ More importantly, the aorta is not only able to dilate under pressure, but its fibers realign to slowly recoil to its initial shape when blood pressure falls.^17^ Therefore, the aorta is not a purely elastic but a viscoelastic material. Hence, the design of synthetic grafts to treat aortic diseases should aim at recovering the vascular wall functions after the intervention.

An interesting yet underutilized way to manufacture viscoelastic materials is electrospinning. Dated back to 1934,^18^ the process consists of exposing a polymer, synthetic or natural, dissolved in a highly volatile solvent, to a high electric potential (10-30 kV) across a finite distance between a conductive needle and a grounded collector.^19^ The solvent evaporates while the polymer flies from the needle tip to the collector and dry fibers deposit on the collector. Advances in electrospinning allow the production of more complex structures,^20^ and the fibers can be collected in various forms such as sheets or tubes for use in engineered skin grafts, blood vessels, heart valves, tendons, and muscles, or as single fibers.^21^

In general, electrospinning can generate fibers as thin as tens of nanometers. Control over the fibers’ diameter and configuration allows for a significant control over the mechanical properties of the graft. Indeed, with the right combination of electrospinning parameters (eg, polymer, solvent, fiber size, alignment, and thickness), it is possible to achieve the desired mechanical properties (ie, stiffness or viscoelasticity).

We hypothesized that an electrospun bilayer microstructure mimicking the aorta’s endogenous viscoelasticity could support endothelial and smooth muscle cells colonization after aortic dissection. Aortyx has ideated a prototype, AX-GEN01, consisting of a patch delivered endovascularly that adheres to the primary entry tear of an aortic dissection thanks to a proprietary cyanoacrylate-based adhesive formulation (Figure 1). After contacting the phospholipids and albumin present in the bloodstream, the adhesive undergoes an exothermic hydroxylation reaction. First, the acidic stabilizer is neutralized, and then the polymerization is initiated with the conjugate addition of anionic nucleophiles, to continue with the propagation phase until full curation. The patch should prevent false lumen perfusion, exhibit mechanical properties similar to the aorta’s, and promote surrounding tissue integration to efficiently treat the dissection, restoring the natural microenvironment to the vessel. This paper focuses on the ability of the adhesive bioresorbable patch to mimic the mechanical properties of the aorta and to promote its integration within the damaged endogenous tissue.

**Figure 1:**
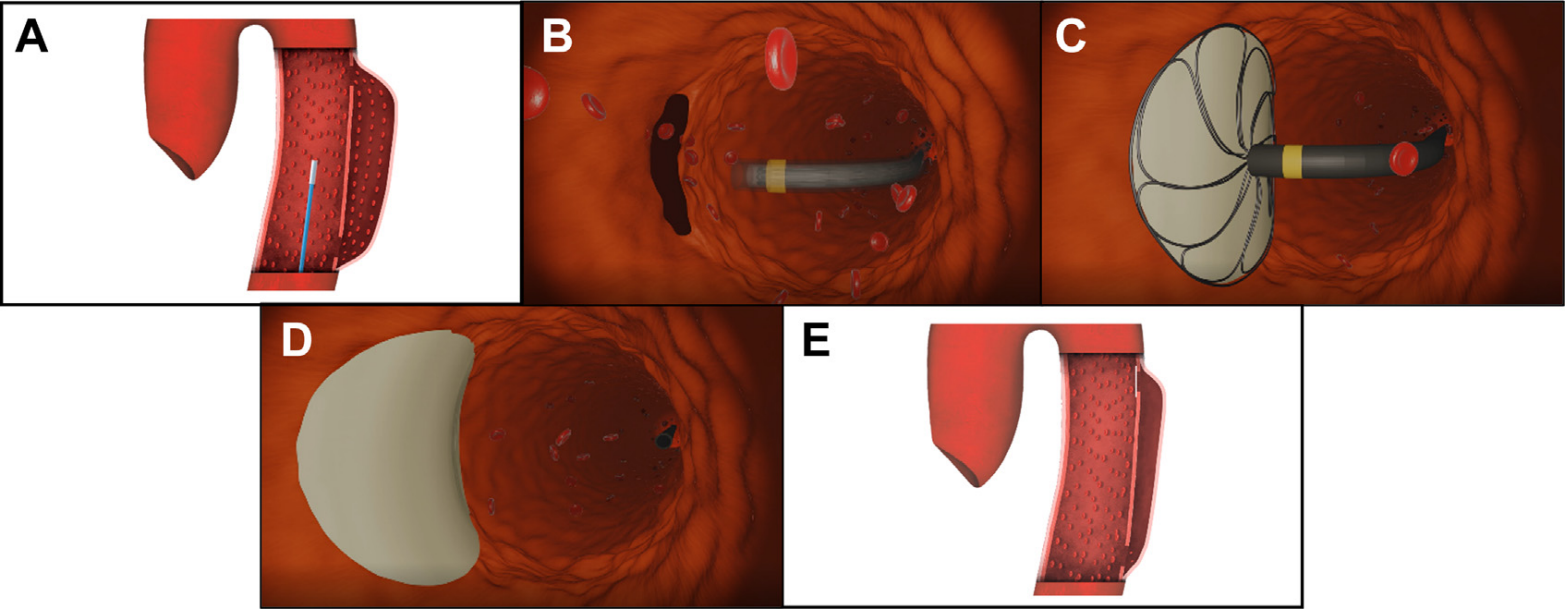
Rationale of AX-GEN01 (A) The steerable catheter navigates with the introducer to the dissected area protecting it from blood. The introducer carries the flower-shaped deployer, which is folded with the patch inside the catheter. (B) Once close to the dissection, the catheter is flexed using the handle and orients the introducer and the patch deployer toward the injury. (C) The deployer opens and exposes the patch to blood. This activates the adhesive in seconds. The deployer applies homogeneous pressure on the patch, which adheres to the artery thanks to the adhesive. (D) After the adhesion time, the soluble stitches rapidly dissolve and release the patch from the deployer. The deployer is retrieved into the catheter, and the system is removed from the body. (E) The patch blocks the entry tear, which deflates because of pressure differential. The false lumen starts the coagulation process.

## Methods

Descriptions of Scanning Electronic Microscopy, MicroCT Scan, mechanical properties measurement, cell culture, biocompatibility, and statistics are available in the Supplemental Appendix, Materials and Methods section.

### Adhesive patch preparation

10-mm circular patches were cut from the electrospun sheets using a die cut. These patches were then deposited on flower-shaped custom Nitinol deployers (Supplemental Figure 1), with the aligned face of the patch in contact with the deployer. The patches were fixed to the deployer using water-soluble stitches manually sutured to the cranial, caudal, left, and right ends of the patch.

The adhesive components were weighed with an analytical balance and mixed with a Speedmixer DAC-330-100SE (FlackTek SpeedMixer). The adhesive was formulated between 1 and 5 days before the testing and stored at 4 °C. 24 μL of adhesive were distributed over the surface of the patch performing a cross in the inner part and a circle in all the perimeter of the 10-mm patch using a positive displacement M10E micropipette (Gilson). Once the adhesive was applied, the time between adhesive application and test was between 1 and 3 minutes.

### Animal studies

Animal experimentation was conducted at Institute Mutualiste Montsouris Recherche (Paris, France). All experiments were conducted in compliance with ISO 10993-2 and with internal Institute Mutualiste Montsouris Recherche’s Standard Operating Procedure “Ethics and Ethics Committee Management.” The Animal Care and Use Committee of the Testing Facility is registered at the CNREEA under the Ethics Committee n°37. The study was assigned to the project ENDOVASCULAIRE 12534. A total of 15 rams were used: n = 3 for 7-day studies, n = 3 for 30-day studies, n = 3 for 90-day studies, and n = 6 for 90-day dissection studies.

In all procedures, the animal was placed in right recumbency to perform a thoracotomy at the 5 to 6 intercostal level under cardiopulmonary bypass. The descending aorta, exposed and horizontally cross-clamped after the arch, was decompressed with removal of blood and stored at room temperature for later use. Once the aorta was emptied, a longitudinal transmural incision of ∼3 cm was made with a scalpel. The adhesive was distributed over the surface of the patch as mentioned earlier, and the patch was implanted perpendicular to the aortic wall using a guiding apparatus that held the patch and deployer in place for 1 minute, applying a constant force of 1 N. After 1 minute, prewarmed (37 °C) saline was poured on the patch for 2 minutes while continuing to apply force to fully dissolve the soluble stitches. The deployer was released, the incision sutured, and blood flow restored. Live follow-up was performed using transesophageal echocardiography (TEE) at different timepoints up to 3 months.

### Histology

After necropsy, fresh tissue was fixed in 4% paraformaldehyde in phosphate-buffered saline at 4 °C for 48 hours. Samples were then dehydrated, processed, and embedded in low melting point paraffin (42-44 °C, Merck Millipore), sectioned (5-7 μm) with a microtome, and stained for hematoxylin and eosin or Masson’s trichrome (MT) where nuclei stain in black; cytoplasm, muscle, and erythrocytes stain in red; and collagen in blue. Images were captured with a Nanozoomer 2.ORS (Hamamatsu) and visualized with NDP.View 2 software. Slides were assessed in the spirit of ISO10993-6 by certified histopathologists.

### Statistical analysis

All in vitro experiments were performed on triplicate specimens and repeated 2 separate times. Normality distribution of the data was tested in all experiments using the Shapiro-Wilk test. When data were normally distributed, pairwise comparisons were performed using Student’s *t*-test, followed by Tukey’s post hoc analysis. In these cases, data are expressed as average ± SE of mean. When data were not normally distributed, pairwise comparisons were performed using Mann-Whitney *U* test. In these cases, data are plotted showing the median with 25th to 75th percentiles. In all cases, values of *P <* 0.05 were considered statistically significant.

## Results

### Microstructure and mechanical properties of the patch

**Figure 2:**
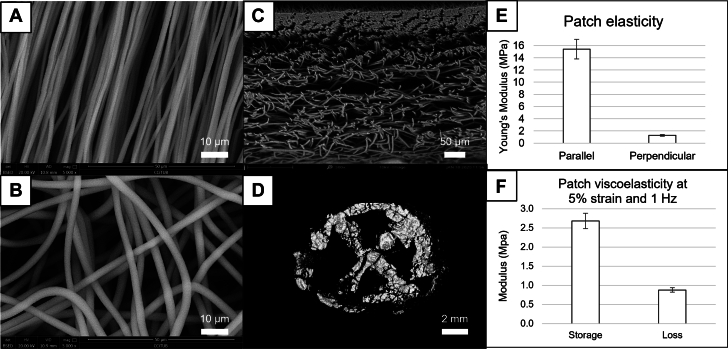
AX-GEN01 Microstructure and Mechanical Properties (A) Luminal face of the patch. The aligned fibers have an average diameter of 1 ± 0.5 μm. They are aligned with flow to promote endothelial cell migration and to stop underlying smooth muscle cell colonization. (B) Abluminal face of the patch. The random fibers have an average diameter of 3 ± 0.5 μm. The high porosity allows for smooth muscle cell colonization and macrophage clustering. (C) Cross-section of the patch. The aligned layer represents approximately 35% of the thickness and the random 65%. (D) MicroCT scan, showing that the adhesive is placed forming a circle and a cross. (E) Young’s modulus of the patch, placed with the aligned fibers perpendicular or parallel to the applied tension. (F) Viscoelasticity of the patch, measured as Loss modulus versus storage modulus at 5% strain. Data are presented as mean ± SEM.

Two-faced patches, made of electrospun polycaprolactone, were manufactured with an average thickness of 180 ± 10 μm. The luminal face consisted of aligned fibers (Figure 2A) with an average diameter of 1 ± 0.5 μm, with more than 90% of the fibers parallel within ±10°. Aligned fibers were designed to align with blood flow, to promote endothelial cell migration and to stop underlying smooth muscle cell colonization. This layer represented 35% ± 5% of the total patch thickness (Video 1). The abluminal face of the patch was comprised of random fibers (Figure 2B) with an average diameter of 3 ± 0.5 μm. High porosity (50% ± 5%) aimed at promoting smooth muscle cell colonization and macrophage clustering. This face accounts for the remaining 65% ± 5% of the total patch thickness (Figure 2C).

The patch was designed to, like the aorta, present different mechanical behavior depending on the direction of property measurement. Indeed, elasticity, expressed as Young’s modulus, was 15.4 ± 1.6 MPa in the direction parallel to the alignment of fibers, but was 1.3 ± 0.1 MPa in the direction perpendicular to fibers (Figure 2E). Coherently, to match with the natural properties of the aorta, the patch should always be placed with the fibers aligned with flow. The viscoelasticity of the patch was also measured in the perpendicular direction, at a 5% strain and cyclic stress of 1 Hz, expressed as storage and loss modulus. In this case, the storage modulus was 2.7 ± 0.2 MPa and the loss modulus was 0.9 ± 0.1 MPa (Figure 2F), for a phase shift of 18° (tan [δ] = 0.34).

### Adhesive and deployer configuration

The deployer force, orientation, displacement, and rotation were combined with the optimum adhesive configuration (formulation, quantity, and disposition) to guarantee attachment to the aorta (Supplemental Figure 2). The adhesive was placed forming a circle and a cross before implantation, a shape preserved after implantation as visualized under MicroCT (Figure 2D). Table 1 shows the mechanical characterization of the adhesive. The most relevant value, adhesion force, was measured as shear strength for a value of 17.8 kPa.

**Table 1:** Adhesive Characterization

Density (kg/m^3^)	1,321 ± 64
Viscosity at 20 °C, 200 s^-1^ (kg/m·s)	1.87 ± 0.12
Surface tension (kg/s^2^)	7.48 × 10^−2^ ± 0.04 × 10^−2^
Curing initialization delay (s)	15.6 ± 2.5
Full polymerization time (min)	6.7 ± 1.4
Shear strength (Pa)	1.78 × 10^4^ ± 0.10 × 10^4^
Lap shear strength (N/mg adhesive)	0.11 ± 0.05
Storage at 4 °C (days)	>90

Of note, the force applied by the deployer is of outmost importance to secure proper patch adhesion to the artery. An applied force of 1 N over a patch of 10 mm in diameter was ideal to guarantee adhesion without forcing adhesive permeation through the patch. Lower forces (0.5 N) did not guarantee sufficient patch-aorta adhesion ex vivo, whereas higher forces (3 and 5 N) caused adhesive permeation and self-attachment to the deployer, preventing efficient patch release. Angles exceeding ±15° of the perpendicular also caused inefficient adhesion of the patch, which led to detachment ex vivo.

### In vitro cellular migration and biocompatibility

**Figure 3:**
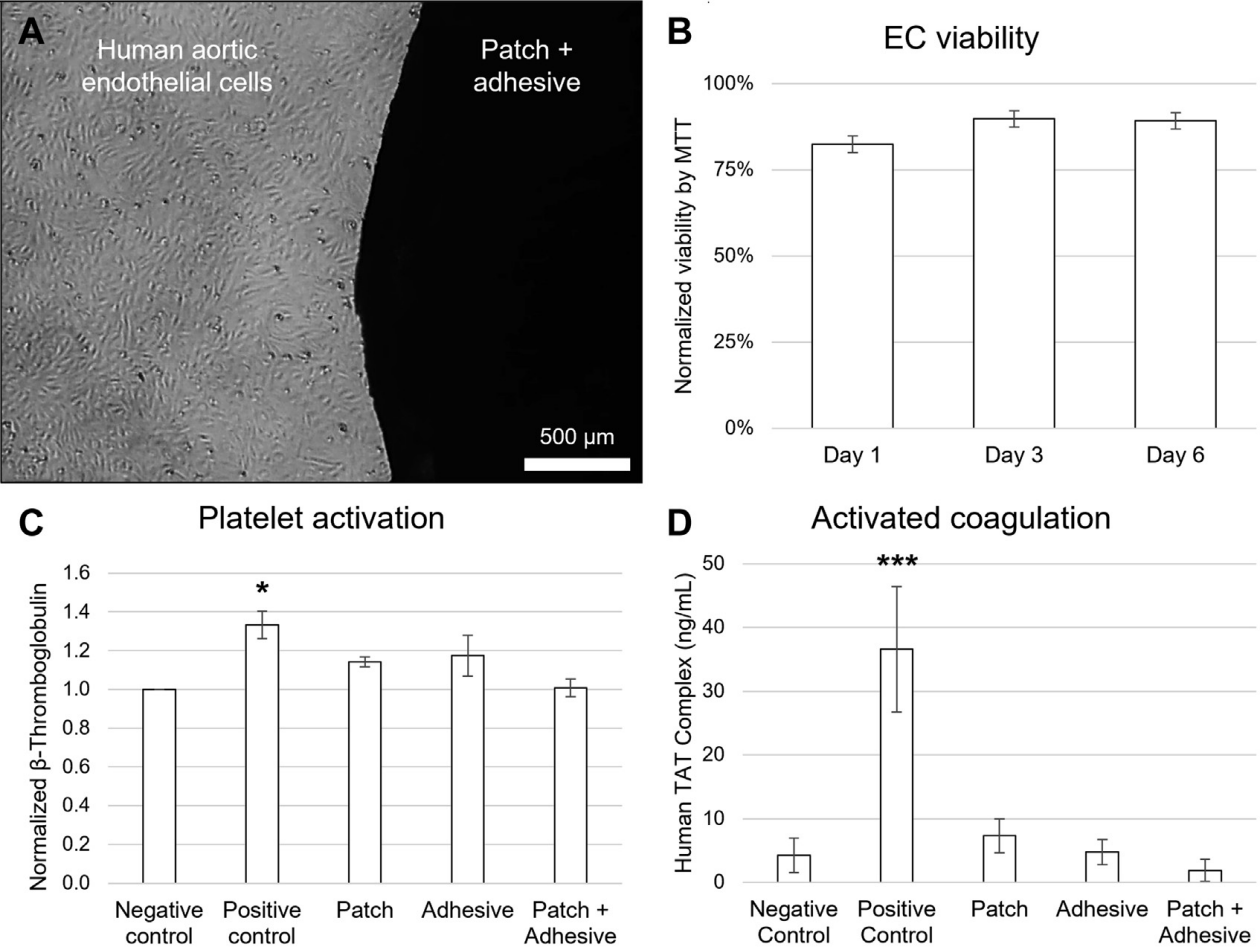
AX-GEN01 Biocompatibility Cytotoxicity and biocompatibility tests for patch, adhesive, and patch + adhesive. (A) EC microscopic shape evaluation migration to the patch + adhesive. (B) Cell viability 3-(4,5-dimethylthiazol-2-yl)-2,5-diphenyl tetrazolium bromide (MTT) test for EC at 1 ,3, and 6 days. (C) Platelet activation using normalized β-thromboglobulin for patch, adhesive, and patch + adhesive. (D) Activated coagulation assay with Human TAT complex for patch, adhesive, and patch + adhesive. Data are presented as mean ± SEM, and pairwise comparisons to the negative control are performed using Student’s *t*-test, followed by Tukey’s post hoc. ∗*P <* 0.05, and ∗∗∗*P <* 0.001.

In vitro cell culture experiments were conducted to demonstrate the patch biocompatibility. Cell viability was assessed for the patch-adhesive system visually and with 3-(4,5-dimethylthiazol-2-yl)-2,5-diphenyl tetrazolium bromide (MTT) every 24 hours for 7 days placing an adherent patch in direct contact with an endothelial monolayer cell culture. Visually, cells in the vicinity of the patch initially acquired a rounder shape, but adapted rapidly adopting hexagonal shape and recovered the space left, with virtually no cytotoxic corona 7 days after implantation (Figure 3A). MTT confirmed the low cytotoxicity of the patch-adhesive ensemble, as no value below 70% (as per ISO 10993-5) was observed at any timepoint, with values ranging between 82% and 89% (Figure 3B). Human β-thromboglobulin and human thrombin-antithrombin levels in the patch alone, adhesive alone, and patch with adhesive systems were measured as platelet and coagulation activation assessment, respectively, but only the positive control was statistically different from the negative control (Figure 3C) (*P* < 0.05) (Figure 3D) (*P <* 0.001).

**Figure 4:**
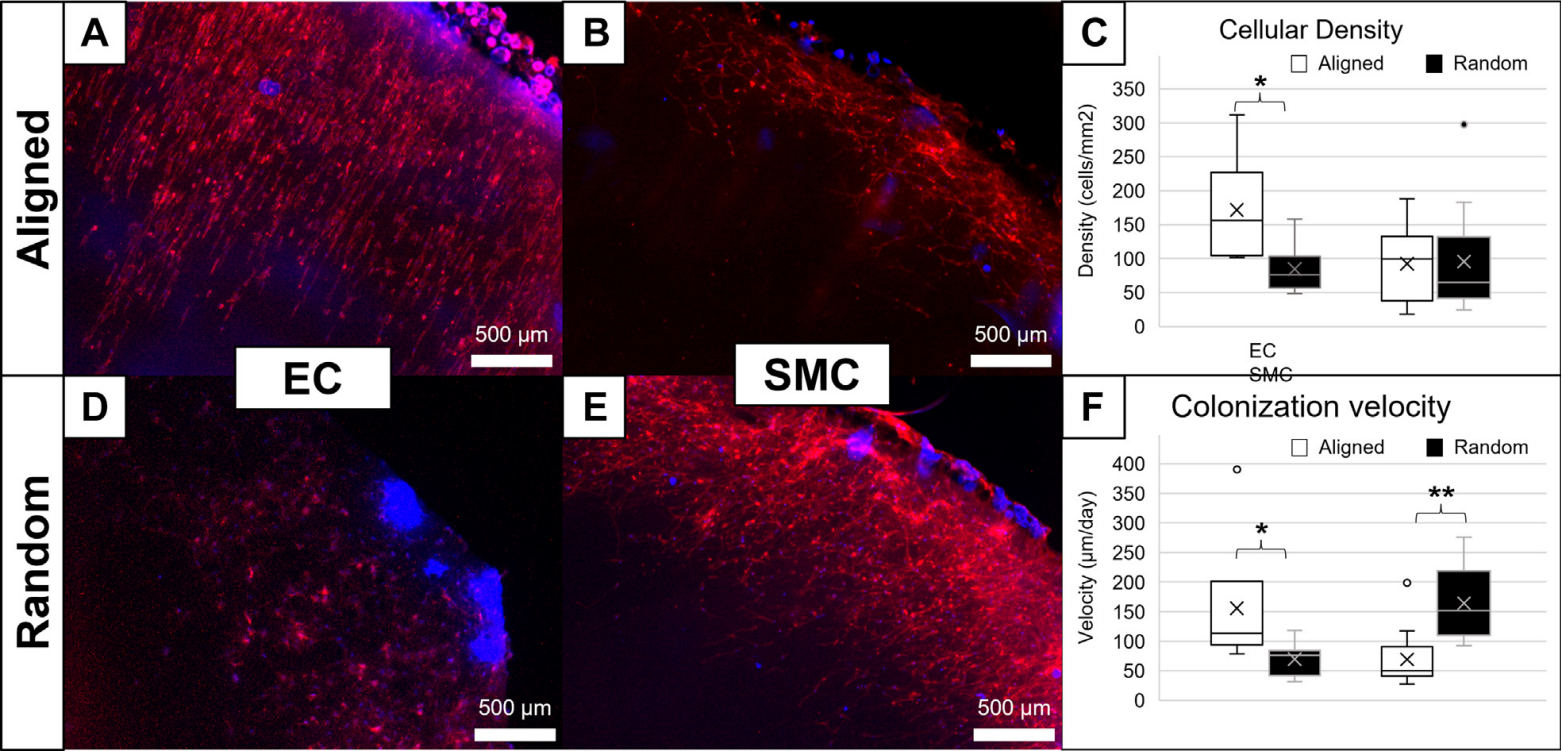
In Vitro Migration Immunostaining and quantification of vascular cells migrating differentially through the patch. Endothelial cell (EC) (A), smooth muscle cell (SMC) (B), and quantification (C) of in the aligned layer, and analogously for the random layer (D, E, F). Cell nuclei are stained in blue (4′,6-diamidino-2-phenylindole) and actin cytoskeleton in red (Phalloidin-Rhodamine). Data are plotted showing the median with 25th to 75th percentiles, and pairwise comparisons are performed using Mann-Whitney *U* test. ∗*P <* 0.05, ∗∗*P <* 0.01.

Next, the differential ability of endothelial cells and smooth muscle cells to migrate in vitro onto the patches was studied. Bilayer patches were glued on tissue culture plastic wells, with the aligned or the random face upwards, and cells were seeded in the vicinity while the patch was covered with a silicone plug to avoid seeding directly on it. The migrative capacity of the cells was evaluated 7 and 14 days later, showing progressive cell coverage of the patch, from the edges to the center (Figure 4). Endothelial cells displayed a healthier, hexagonal morphology and higher density in the aligned layer than in the random (172 ± 46 cells/mm^2^ vs 85 ± 20 cells/mm^2^; *P* < 0.05) and migrate faster (155 ± 67 μm/day vs 69 ± 16 μm/day; *P* < 0.05). As foreseeable, endothelial cells aligned their structure with the patch fibers. Smooth muscle cells displayed similar density in both faces (92 ± 30 vs 96 ± 63 cells/mm^2^; *P =* 0.66), but a healthier, spindle-shaped morphology in the random layer than in the aligned. They also migrated faster in the random layer than in the aligned (69 ± 25 μm/day vs 164 ± 47 μm/day; *P* < 0.01). Together, these results show that neither the patch nor the adhesive compromise cell viability and that the bilayer differential microstructure of the patch allows for differential cell colonization.

### In vivo chronic performance in healthy rams

**Figure 5:**
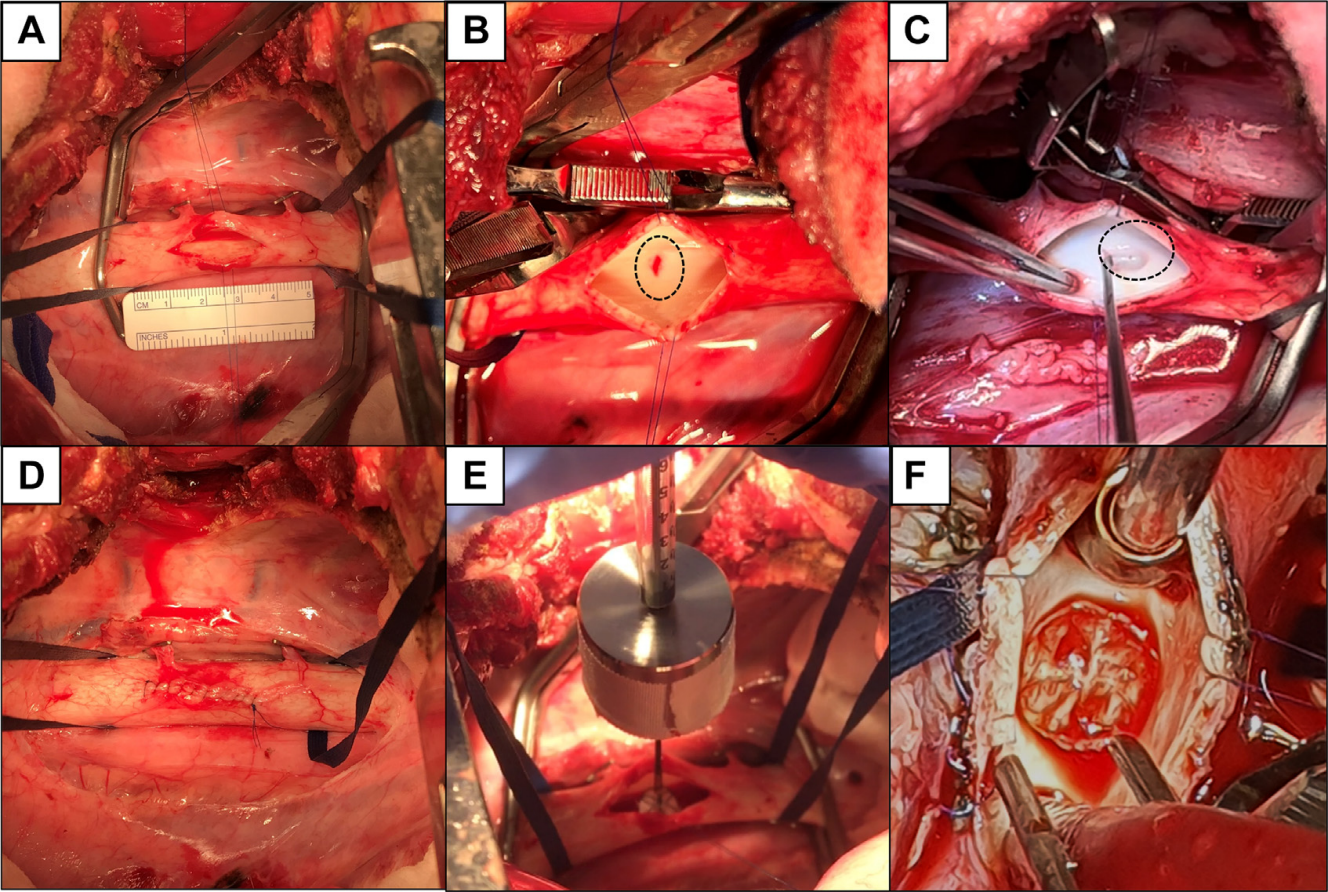
Patch Implantation In Vivo on Rams (A) The aorta is exposed at the 5th to 6th intercostal, is cross-clamped, and an incision is made with a surgical blade. (B) An intima-media tear is created using a surgical blade. (C) The false lumen is expanded using a nerve’s hook. (D) The exposure incision is sutured, and flow is temporarily re-established to extend the false lumen. After that, the aorta is cross-clamped again, emptied and, opened. € A deployer with a weight of 100 g (0,981 N ≈ 1 N) is used to apply homogeneous force on the patch. (F) The patch is left implanted, the artery is closed, and circulation is re-established. In the cases where no dissection was created, steps B, C, and D were skipped. Dashed circle represents the place where the tear is made.

In vivo adhesion and resistance to flow, as well as physiological response (inflammation and thrombogenicity), were assessed in healthy rams (Figure 5). Animals were anesthetized and were put under cardiopulmonary bypass; the aorta was exposed, cross-clamped, and transected; and 10-mm patches were implanted. After implantation, flow was re-established, and animals were awakened.

**Figure 6:**
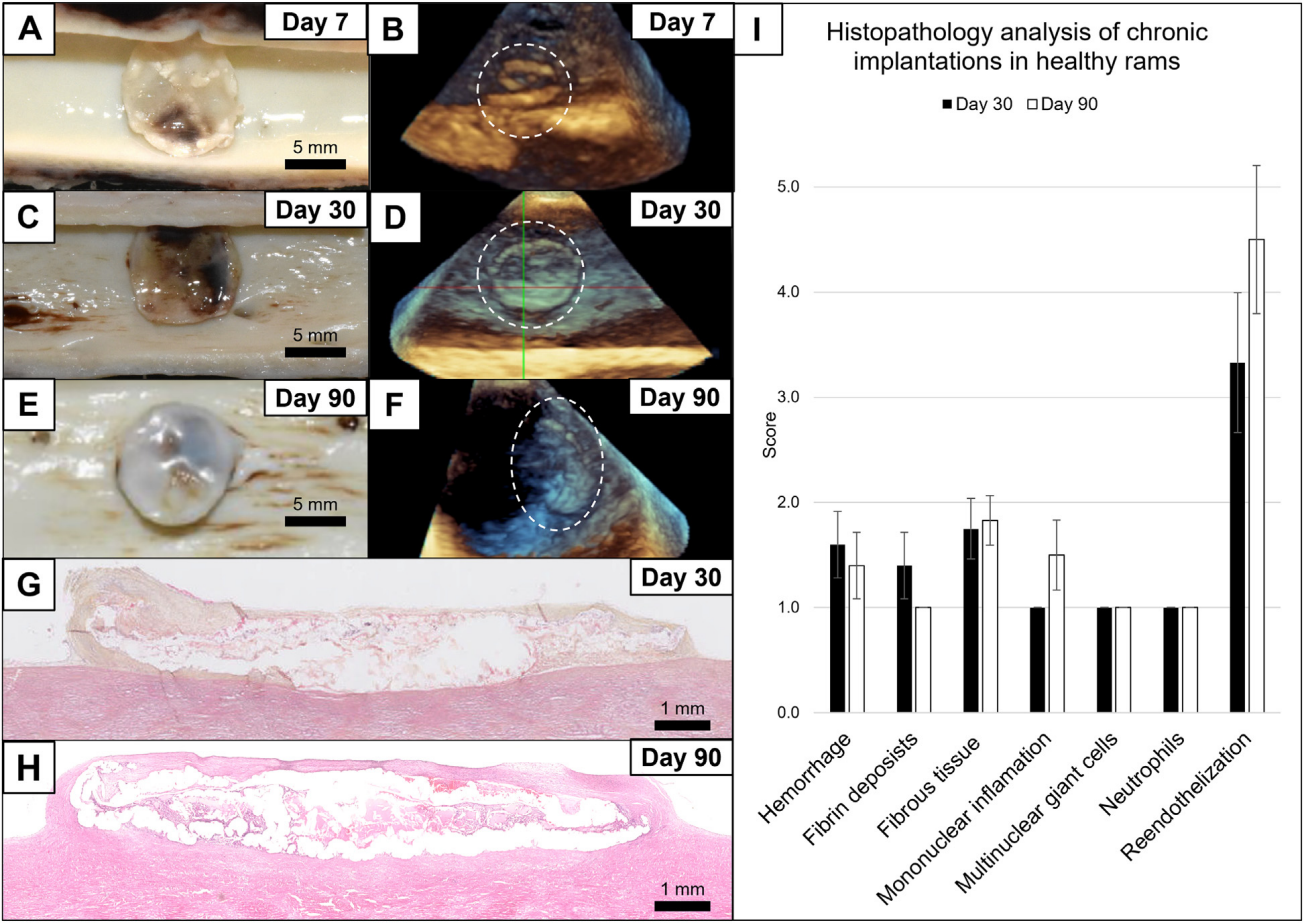
In Vivo Results in Intact Aortae Necropsy at 7 (A), 30 (C), and 90 (E) days postimplantation of representative healthy rams. Transesophageal echocardiography before animal sacrifice at 7 (B), 30 (D), and 90 days (F) postimplantation of representative rams. The images demonstrate how the progressive tissue integration of the patch observed at necropsy can be visualized without sacrificing the animals using transesophageal echocardiography. Hematoxylin and eosin staining of the transversal sections of 30 days (G) and 90 days (H) postimplantation. The left is the cranial and the right is the caudal. (I) Histopathological scores of 1 mean null or minimal and 5 mean high. Data are presented as mean ± SEM.

All animals survived surgery in good clinical condition. Live follow-up was performed using TEE every 7 days and at sacrifice. Patches were clearly identified during in vivo monitorization with TEE, thanks to their intense echogenicity (Figures 6B, 6D, and 6F). The attached patches pulsed following aortic motion at every cardiac cycle, consistent with a mechanical coherence between the implant and the vessel (Video 2). Over time, a loss of patch signal was observed, indicative of the integration process of the patch in the local tissue. These findings were confirmed at necropsy and after histological analysis after 7, 30, and 90 days (Figures 6A, 6C, and 6E).

At 30 days, the luminal layer of the patch was overall covered in all sections. A fibrous layer, thicker on both edges, was multifocally present above the patch and between the patch and the intimal surface of the aorta, indicating consistent integration (Figure 6G). The fibrous tissue above the patch, on the luminal aligned face, was partially endothelialized (average score 3.3 of 5). There was minimal to null mononuclear inflammation (average score 1 of 5) and multinucleated giant cells (average score 1 of 5) within the fibrous tissue and at the surface of the luminal layer of the patch. Overall, inflammation was considered minimal. Minimal signs of hemorrhage (average score 1.6 of 5) and fibrin deposits (average score 1.4 of 5) were also observed.

At 90 days, the luminal layer of the patches was overall almost completely endothelialized (Figure 6H) and only occasionally multifocally interrupted (average score 4.5 of 5). A mild fibrous tissue, completely endothelialized, covered the luminal surface of the patch at both the cranial and caudal edges, and was thicker on both lateral edges. There was minimal to null mononuclear inflammation (average score 1.5 of 5) or multinucleated giant cells (average score 1 of 5) within the fibrous tissue and in contact with the adhesive material, consistent with slow degradation dynamics of the adhesive bioresorbable patch. Overall, inflammation was considered minimal. Hemorrhage (average score 1.4 of 5) and fibrin deposits (average score 1 of 5) were also minimal to null. Local tolerance of the patch and adhesive was considered excellent, with very good tissue integration, fibrous tissue maturation, excellent local tolerance, null thrombogenicity. and initial signs of bioresorption at 3 months (Figure 6I).

### In vivo performance in a dissection model in rams

A similar experiment was performed in 6 rams, but this time, an intima-medial tear mimicking a dissection was created after clamping the artery. After unclamping, blood flow was allowed through the tear to create a false lumen. One animal was sacrificed within the first 24 hours postimplantation because a secondary dissection was created caused by excessive manipulation during the procedure. This dissection progressed distally, causing lower limbs and spinal ischemia, inevitably requiring protocol-mandated euthanasia. Very interestingly, necropsy showed that the secondary, unintended dissection provoked the damage in the animal, not the intentionally caused and patched intima-medial tear, which had thrombosed (Supplemental Figure 3). The remaining 5 rams survived in good clinical condition until sacrifice at 90 days.

During ultrasonic follow-up, rapid thrombosis and collapse of the false lumen was observed, and no dissection was observed in any animal 7 days after patch implantation. Also, the patch signal was progressively lost, consistent with previous experiments in healthy rams without a tear.

**Figure 7:**
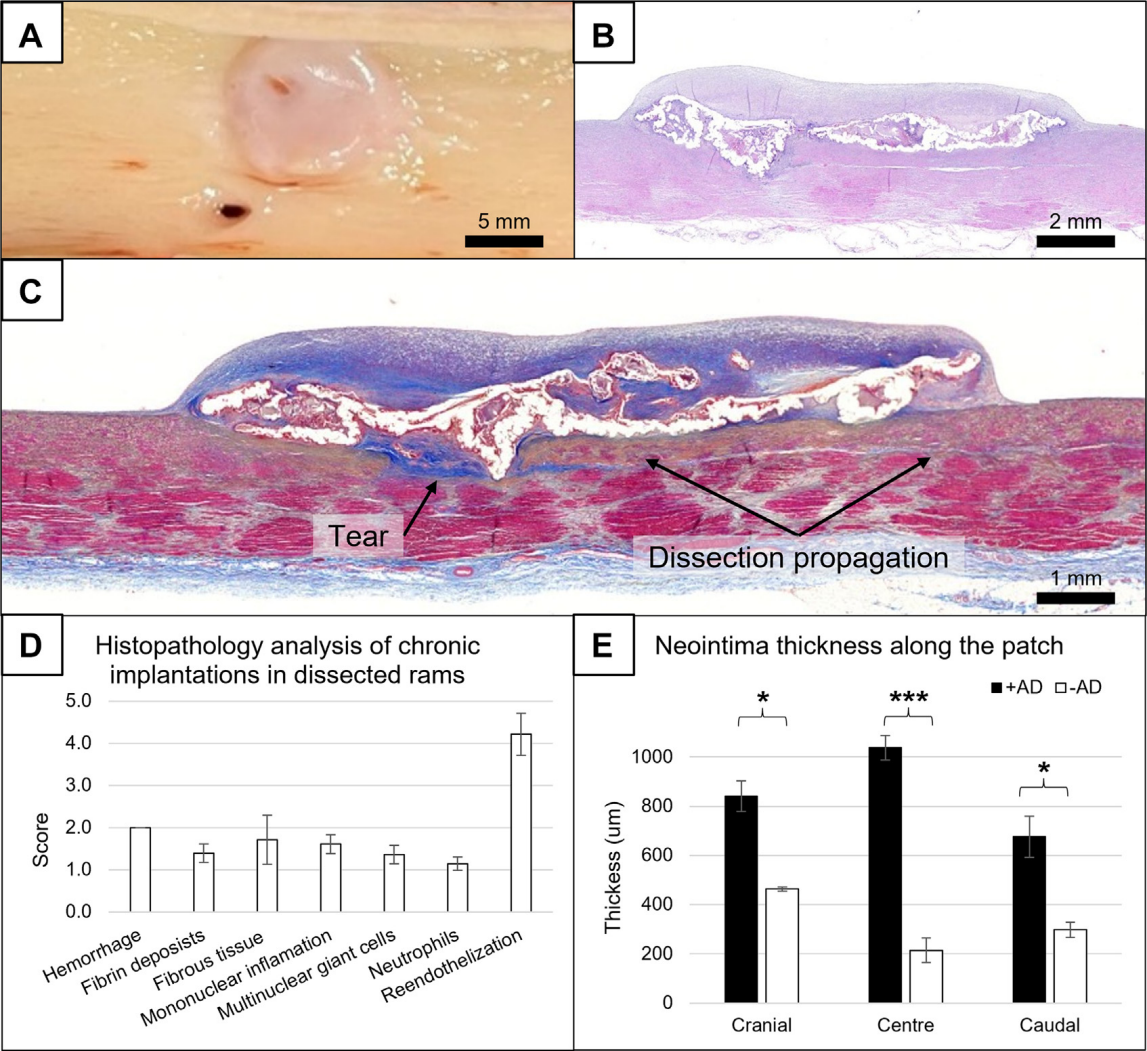
In Vivo Results in a Model of Aortic Dissection (A) Necropsy at 90 days postimplantation of representative dissected rams. Hematoxylin and eosin (B) and Masson’s trichrome (C) staining of consecutive sections at 90 days postimplantation. Arrows indicate tear location and dissection propagation. (D) Histological analysis postimplantation. (E) Neointima thickness comparison between the response in healthy vs dissected aortae. +/- AD represents with or without surgically created aortic dissection. Data are presented as mean ± SEM, and pairwise comparisons are performed using Student’s *t*-test, followed by Tukey’s post hoc. ∗*P <* 0.05, ∗∗∗*P <* 0.001.

At necropsy, all patches were in place, and no visible thrombosis was observed in any of the samples. One animal had pancreatitis, most likely caused by the pharmacological load, and another one had pelvic artery distension. No correlation between the procedure and these events was found. In 4 animals, the luminal layer of the patches was overall almost completely endothelialized (Figure 7A) and only occasionally multifocally interrupted (average score 4.2 of 5). In one animal, this fibrous cap was not present, although significant neointima was observed in the edges of the patch.

A mild fibrous tissue, completely endothelialized, covered the luminal surface of the patches attached to dissected rams (Figure 7B). There was minimal to null mononuclear inflammation (average score 1.6 of 5) and multinucleated giant cells (average score 1.4 of 5) within the fibrous tissue and in contact with the adhesive material, consistent with slow degradation dynamics. Overall, inflammation was considered minimal. Hemorrhage was slightly higher than in healthy rams (average score 2 of 5), consistent with the damage made during the provoked dissection, and fibrin deposits (average score 1.4 of 5) were also minimal. Local tolerance of the patch and adhesive was considered excellent, with very good tissue integration, fibrous tissue maturation, excellent local tolerance, null thrombogenicity and initial signs of bioresorption at 3 months (Figure 7D). Of note, the neointima in dissected rams was significantly thicker in all segments of the patch (*P <* 0.05 in the cranial and caudal segments; *P* < 0.001 in the center) than in animals without an artificial injury created (Figure 7E, Supplemental Figure 4).

Interestingly, Masson’s trichrome staining showed significant maturation of the fibrous tissue overlaying the patch. Most excitingly, the region where the dissection was generated was clearly identified (Figure 7C), presenting a fibrous tissue that covered the entry tear. The entire dissection caudally to the entry tear presented as blue staining, characteristic of fibrous tissue, confirming compression and remodeling of the artificially created false lumen.

## Discussion

We demonstrate, in vitro, ex vivo, and in vivo, the potential of an electrospun adhesive bioresorbable patch to be used as a treatment for aortic dissections.

Electrospinning is a technique that has enormous potential for medical devices. Indeed, it is already present in approved products like Medprin ReDura, Zeus bioweb, or ZB Biowick.^22^ The technique, however, presents certain manufacturing limitations in consistency and reproducibility, because fibers fly off randomly to land on a rotating drum. Major medical device manufacturers have intentionally avoided its use because of the difficulty in interbatch and intrabatch reproducibility. If reproducibility is achieved, then electrospinning can, as we have demonstrated, tailor the mechanical properties of polymeric sheets to the needs of the target tissues.

Although the use of bioresorbable polyesters in external applications is common, some polyesters have failed in the past in vascular applications. The most infamous case is the U.S. Food and Drug Administration’s decision to withdraw Abbott’s bioresorbable coronary stents due an increase in very late stent thrombosis.^23^ Unfortunately, this has tainted the evolution of bioresorbable devices in endovascular environments for reasons not strictly related to the nature of the material. The stents pulled from the market were made of polylactic acid, whose inherent material properties coupled with the required strut thickness created a nonporous and extremely stiff material,^24^^,^^25^ unlike our device. Several authors^26^, ^27^, ^28^ have pointed out that the microrecirculation downstream of the stent strut is likely to be the cause of such thrombosis, in particular when the stent strut (≈200 μm) occupied significant space in the already narrow and compromised lumen of atherosclerotic arteries. This is definitely not the case in the Aortyx patch. This patch is designed for application in the aorta, a much larger artery where such microrecirculations are not impactful and would not cause significant thrombosis because of the elevated flow and diameter of the vessel. Our in vivo and ex vivo results show no thrombogenicity. Furthermore, the patch is extremely porous, promoting a much better tissue integration that also prevents from late device thrombosis given that the patch is soon covered by non-thrombogenic endothelium.

The aorta should not be considered only a passive conduit for the bloodstream,^29^^,^^30^ but also as an elastic reservoir, enabling the arterial tree to undergo large volume changes with little pressure changes.^31^ The viscoelasticity of the aorta is a critical yet overlooked physical property that should be considered when designing new biomaterials for aortic treatments. Matching the aorta’s mechanical properties is critical to avoid mechanical mismatches, and consequent device failure. In that sense, the literature is extremely broad. It is not straightforward to find the right mechanical characterization because most studies remove the aorta from its natural setup, and this significantly tampers the measurements. The most consistent studies present radial Young’s modulus between 0.5 and 3.0 MPa in human healthy aortas, and this was our goal range to design our patch.^32^, ^33^, ^34^, ^35^, ^36^ Data on viscoelasticity is even more sparse and rare, but our data are consistent with recent literature.^10^^,^^37^, ^38^, ^39^ Our echocardiographic measurements validate that indeed we are in the range of natural tissue properties, because the patch is fully compliant with the natural motion of the aorta, but the exact match with the tissue’s mechanical properties is hard to guarantee.

We have described an adhesive bioresorbable patch able to promote vascular cells colonization with minimal to no inflammation and thrombogenicity up to 90 days in large animals. Nonetheless, like all studies, our work presents limitations. Although our goal is to develop an endovascular repair patch, we have used an open approach to implant our patches. In the human ascending aorta, the risk of dissection is significant after the diameter exceeds 45 mm for Marfan syndrome patients and 50 mm for patients without detected genetic alterations.^40^^,^^41^ Only extremely large animals, not fit for animal research facilities, present aortae that large. Coherently, to prove that the patch works independently of the endovascular delivery system, we decided to use large (90 kg) rams with aortae in the range of 15 to 20 mm. Rams are a common model for research on the cardiovascular system and are excellent for chronic studies because they only grow until they reach adult age, whereas pigs continue to gain weight over time.^42^^,^^43^ Importantly, we had to reduce the patch diameter from 30 mm (for human applications) to 10 mm to guarantee that the ratio of patch diameter to artery perimeter was maintained.

The adhesive has a shear strength about 10,000 times superior to the shear caused by blood flow^44^^,^^45^ in the aorta. We applied the adhesive manually using a micropipette that creates a specific pattern. This is undoubtably far from a real clinical application because the risk of inadequately distributing the adhesive is too high, potentially leading to patch detachment.

### Study Limitations

We have created an ad hoc dissection model for our study, inspired by other published dissection models.^46^, ^47^, ^48^ Aortic dissections are naturally originated in a biological microenvironment that is characterized by elevated matrix metalloproteases,^49^ altered TGF-β pathway,^50^ and proinflammatory conditions.^51^ Our surgically created tear does not recapitulate the physiopathology of the dissection, but the physical lesion provoked an increased tissue ingrowth and patch encapsulation, compared with attaching the patch onto healthy aorta tissue.

Perspectives **COMPETENCY IN PATIENT CARE AND PROCEDURAL SKILLS:** We demonstrate the power of using a biomimetic scaffold in a vascular environment We foresee a rapid adoption by vascular surgery and cardiothoracic surgery professionals after clinical trials are successful. Initial clinical research will focus first on descending aorta dissection but will rapidly evolve towards the most life-challenging cases in the ascending aorta.

**TRANSLATIONAL OUTLOOK:** Endovascular repair of aortic dissection still presents very significant limitations. Preserving the mechanical and biological properties set by the aortic microstructure is critical to the success of implantable grafts. In this paper, we present the performance of an adhesive bioresorbable patch designed to cover the entry tear of aortic dissections.

## Conclusions

A novel bioresorbable adhesive patch to treat aortic dissections has been developed and validated in vivo. The patch does not present biocompatibility issues and initiates a natural integration in the tissue. Further studies in humans are necessary for its final validation.

## Funding Support and Author Disclosures

This work was supported by the SME Phase 1 by the European Commission (Grant Agreement 876061); Llavor, Producte, FI and Doctorat Industrial by Generalitat de Catalunya (2016-LLAV-00061, 2018-PROD-00134, 2020FI-B00675 and DI-60-2020); Neotec by Centro de Desarrollo Tecnológico Industrial (SNEO-20191077); Programa Torres Quevedo by MINECO, and EU NextGenerationEU/PRTR program (PTQ2020-011126 MCIN/AEI/10.13039/501100011033); CaixaImpulse and CaixaImpulse Consolidate by Fundació Obra Social “la Caixa” (CI17-0022 and CI92-00003), and Headstart by EIT Health (Spain2019-HS-0072); and R01 HL161069 by National Institutes of Health (to Dr Edelman). Drs Balà, Aranda, Teixidó, Molhoek, Moreno, Febas, López-Guimet, Borrós, Martorell, and Riambau are or have been employees and/or hold stock from Aortyx SL, the company that aims to use this invention. All other authors have reported that they have no relationships relevant to the contents of this paper to disclose.

## Appendix

Video 1 3D Reconstruction of the Patch 3D reconstruction of the patch surfaces, showing the differential layers and the thickness ratio aligned vs random.

Video 2 Implanted Patch TEE Pulsatility of the patch in concordance with the natural movement of the aorta as seen under 3D transesophageal echocardiography.
