# Supplementary material for: In Vivo Efficacy of an Adhesive Bioresorbable Patch to Treat Aortic Dissections
Source: JACC Basic Transl Sci. 2023 Oct 11;9(1):65–77. doi: 10.1016/j.jacbts.2023.08.002 (PMC10864981; doi:10.1016/j.jacbts.2023.08.002)

## Supplementary Materials and Methods

### Patch electrospinning

The electrospinning apparatus (FluidnatekTM, LE-10) was set up horizontally. Polymer solution was loaded in a 5 mL Luer lock plastic syringe and it was pumped through the syringe needle of 0.8 mm diameter. The syringe was placed on a syringe pump system and the mixture was propelled at a volumetric flow of 2,000 µL/h. The syringe pump was placed horizontally, 18.0 cm away from the collector. A voltage potential from 16.0 to 20.0 kV was applied between the syringe pump and the collector. The collector used was the drum of 10.0 cm in diameter. The mixture was deposited on the collector for 2 hours. For the first hour the drum was maintained static and for the second hour it was rotated at a 1,000 rpm speed (clockwise direction). The patch was afterwards air dried for approximately 1 hour.

### SEM

Fiber morphology and patch thickness were evaluated with a scanning electron microscope (Quanta-200 SEM). The samples were not coated, and the images were taken with low vacuum and a back scattered electron detector (BASED). ImageJ was used to measure the fiber diameter and thickness.

### Microscopic computerized tomography scanning (MicroCT Scan)

X ray scanning was performed using a SkyScan 1172 microCT (Bruker MicroCT, Belgium). The excised aorta fragment was wrapped with gauze soaked in PBS, placed in plastic container and sealed to avoid evaporation. Samples were scanned with identical settings: X‐ray source 50 kV, 500 μA, exposure time 1250 ms, averaging (2), average voxel size of 13 μm, rotation step 0.5° over a range of 180 degrees. After reconstruction with NRecon Recon software (version 1.6.8.), all the serial section grayscale images were set at a threshold to identify adhesive. The binarized 3-dimensional images were visualized with CTVox (version 2.4) and quantified with CTAn (version 1.18).

### Mechanical properties patch and adhesive

Geometry

Electrospun sheets were cut into long, thin, rectangular (30 mm length and 5 mm width) strips. The strips were cut in two different directions: parallel to the aligned fibers or perpendicular to the aligned fibers. Width and thickness were measured at three locations along the length of each sample using an electronic digital caliper (Fervi, Modena, Italy), averaged, and recorded.

Patch elasticity

Patch stiffness was expressed as Young’s modulus and determined via uniaxial stress-strain tests performed using a dynamic mechanical analysis machine, DMA Q800 (TA instruments, Newcastle, DE, USA) and TA Universal Analysis 2000 software. 1 cm in length and 0.5 cm in width samples were preloaded at 0.01 N with an initial strain of 0.9%. Samples were then constantly strained at 5.0 % per minute until reaching a displacement of 30.0 % from the initial length.

Patch viscoelasticity

Patch viscoelasticity was measured using the DMA Q800. 1 cm in length and 0.5 cm in width samples were preloaded at 0.03 N, and an initial strain of 0.1%. Samples were cyclically strained at 1 Hz, exposed to a growing amplitude of 5 to 200 μm. Storage and loss modulus were evaluated at a strain of 5%, and the phase shift was calculated as the arctangent of the loss modulus divided by the storage modulus.

Adhesive shear strength

The strength of the adhesive formulation was determined by the lap shear test according to ASTM F2255-05 and the tissue used for the assay was pig aorta obtained from the butcher.

Adhesive curing time

The curing time of the adhesive formulation was determined by direct contact of the formulation with a Fetal Bovine Serum (FBS) bath at 37 ºC. The adhesive was applied to a polyethylene film and subsequently immersed in the FBS bath. The curing time was measured visually, since the adhesive changes color as it polymerizes.

### In vitro testing

Cell Culture

Human aortic endothelial cells (EC) and smooth muscle cells (SMC) were acquired from Promocell (Heidelberg, Germany) and cultured in EBM-2 basal medium (C-22111, Promocell) supplemented with 0.02 ml/ml fetal bovine serum (FBS), 1% penicillin-streptomycin, 0.2 μg/ml hydrocortisone, 22.5 μg/ml heparin, 10 ng/ml hFGF-B, 0.5 ng/ml VEGF, 20 ng/ml R3-IGF-1, 1 μg/ml ascorbic acid and 5 ng/ml hEGF (EGM-2), or SBM-2 basal medium (C-22162, Promocell) supplemented with 0.05 ml/ml FBS, 1% penicillin-streptomycin, 0.5 ng/ml hEGF, 2 ng/ml hFGF-B and 5 μg/ml insulin. Cells were fed every 2-3 days and incubated at 37 ºC and 5% CO_2_ in a humidified incubator. Experiments were performed using cells in passages ranging from passage 4 to 6.

Cytotoxicity

Following the spirit of ISO10993-5, direct contact method, EC passage 4-6 were seeded to confluence on 60 mm diameter tissue culture dishes. The patch was adhered onto the center of the tissue culture plate. The ensemble was incubated at 37 ºC and 5% CO_2_ in a humidified incubator for 24, 48, 72, 96, 144 and 168 h. The samples were examined microscopically using a contrast microscope, with special focus on the perimeter of the patch-adhesive system, and cellular viability was evaluated via MTT assay (Merck Millipore).

Cellular Migration

10 mm patches were adhered to the bottom of 6-well tissue culture plates and covered with a silicone plug to prevent cell attachment during cell seeding. Cells were seeded on the tissue culture plate and, after 24 hours of incubation at 37 ºC 5% CO_2_, the silicone plug was removed, and cells were allowed to proliferate and spread on the patch surface for up to 14 days. At each timepoint, patches were detached from the wells and cells were fixed with 4% PFA and stained with Phalloidin (1:100 in PBS, Merck), and DAPI (1:100 in PBS, Merck). The distance between the edge of the patch and the furthest cell was measured and averaged for every timepoint.

Thrombogenicity

Following the spirit of ISO10993-4 and ASTM F2888-13, patch, adhesive and patch + adhesive were cut in 1 cm in length x 2 cm in width pieces and placed in a tube containing 1 mL of fresh blood recalcified and heparinized right before the experiment. The patch with adhesive was placed at the bottom of the tube to only expose the aligned parted of the patch to blood, and samples were left in the incubator for 1 hour at 37ºC and 60 rpm.

Immediately after incubation, 100 μl were aliquoted to count platelets using a platelet counter (PC100, DMD, Netherlands). 400 μL were aliquoted and resuspended in 0.106 M Sodium Citrate, and β-thromboglobulin (β-TG) was measured using a human β-thromboglobulin ELISA kit (MyBioSource, Canada), and normalized by number of platelets. 400 μL were aliquoted and the human thrombin-antithrombin complex (TAT) was directly measured using its ELISA kit (Abcam, Cambrdige, MA). All tests included a negative (fresh blood) and a positive (EPDM black rubber) reference material.

## Supplementary Figures

Figure S1: A) Patch attached to the deployer before adding the adhesive. B) Adhesive attached in a cross + circle shape. C) Pressure applier that homogeneously applies 0.98 N on the deployer and the patch. D) Patch implantation *ex vivo* on a porcine aorta. E) Implanted patch.


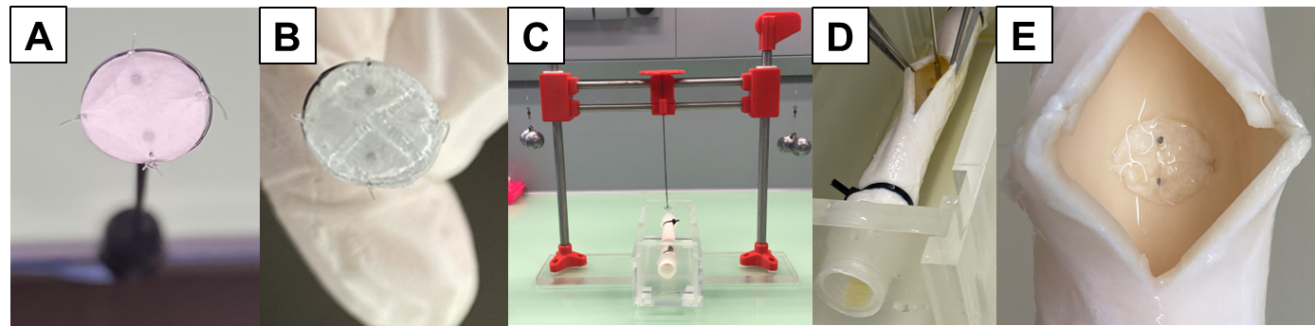


Figure S2 – Deployer characterization: Ink tests allow to track the force distribution on the patch. In conditions squared in green, the force is homogeneously applied on the perimeter. In conditions squared in red the test fails as the perimeter is discontinued.


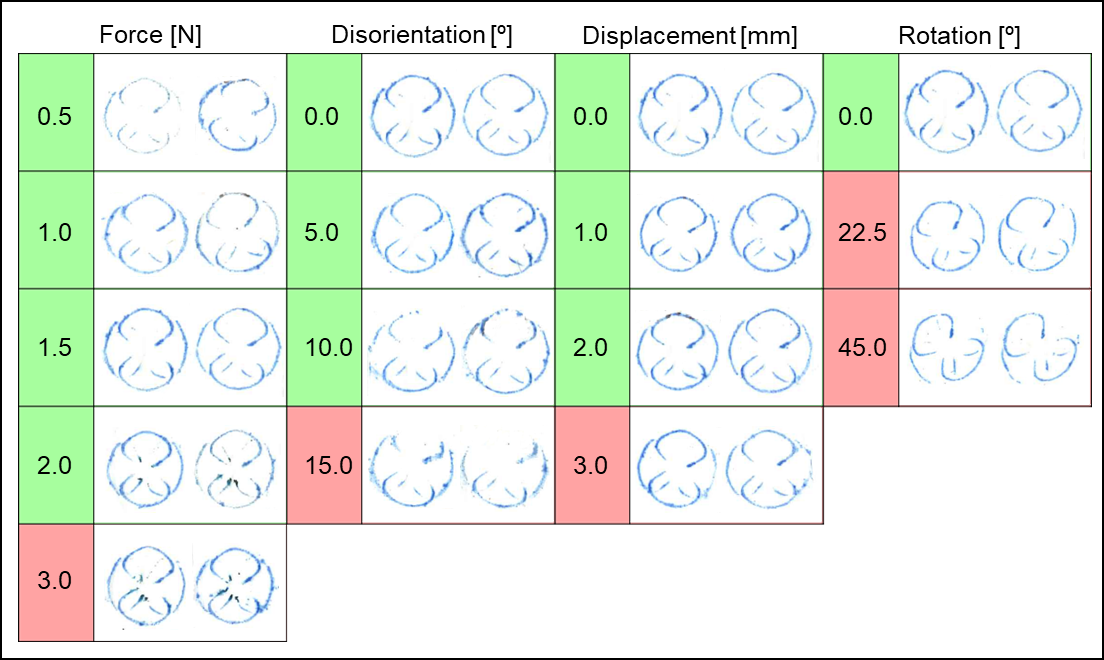


Figure S3 – Fully dissected aorta:

In one of the experiments performed, the dissection created propagated caudally excessively, causing limb ischemia in the specimen obliging, per ethics protocol, to sacrifice the animal 18 hours after the implantation. A) In the TEE, a large false lumen is clearly identified under the patch. B) Necropsy post-sacrifice, showing the extent of the dissection. C) H&E histology of the dissection, confirming the extent, and that the area under the patch was fully thrombosed 18h post-implantation.


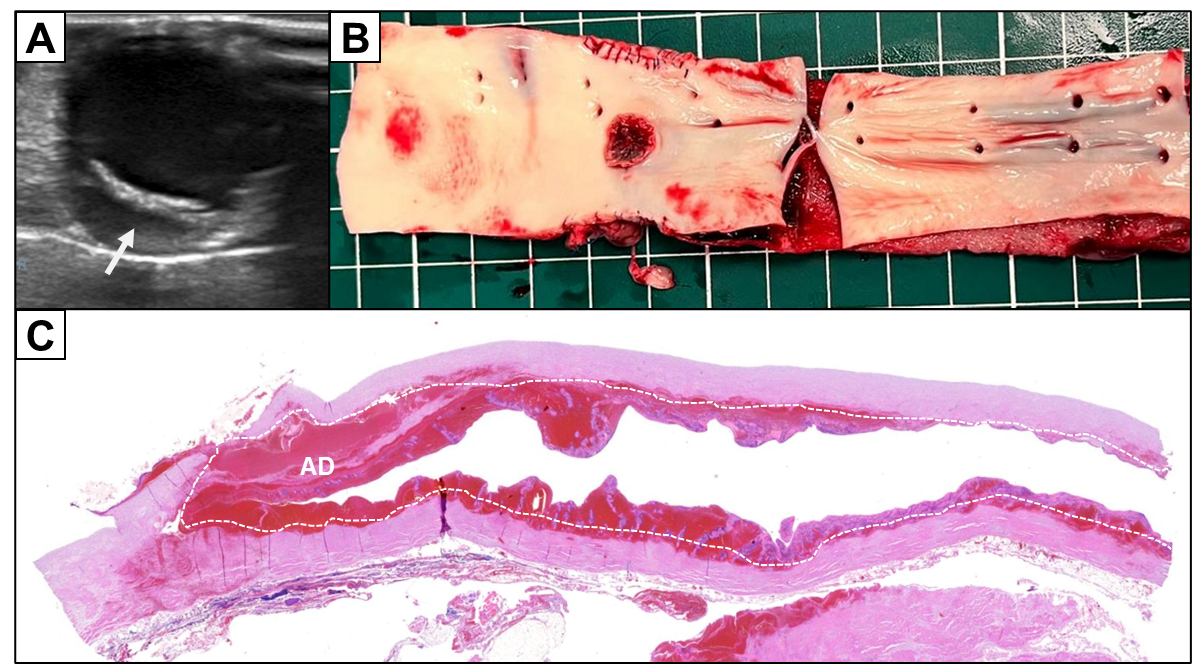


Figure S4 – Neointima thickness: Neointima at 90 days with and without dissection. The thickness is measured at 25% (cranial), 50% (central) and 75% (caudal) as seen in the figure.


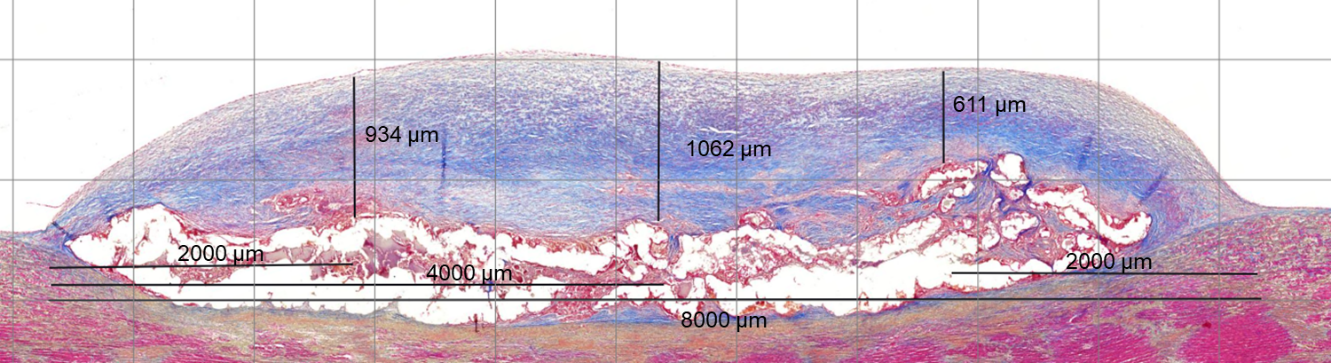

Supplement: Supplemental Methods and Supplemental Figures 1-4 [file mmc3.docx]
